# Productivity and Nutrient Balance of an Intensive Rice–Rice Cropping System Are Influenced by Different Nutrient Management in the Red and Lateritic Belt of West Bengal, India

**DOI:** 10.3390/plants10081622

**Published:** 2021-08-06

**Authors:** Tanmoy Shankar, Ganesh Chandra Malik, Mahua Banerjee, Sudarshan Dutta, Sagar Maitra, Subhashisa Praharaj, Masina Sairam, Duvvada Sarath Kumar, Eldessoky S. Dessoky, Mohamed M. Hassan, Ismail A. Ismail, Tarek Saif, Milan Skalicky, Marian Brestic, Akbar Hossain

**Affiliations:** 1 Department of Agronomy, Centurion University of Technology and Management, Odisha 761211, India; tanmoy.shankar125@gmail.com (T.S.); sagar.maitra@cutm.ac.in (S.M.); subhashisa.praharaj@cutm.ac.in (S.P.); sairammasina52@gmail.com (M.S.); sarathkumarduvvada010@gmail.com (D.S.K.); 2 Department of Agronomy, Palli Siksha Bhavana, Visva-Bharati, Sriniketan 731204, India; ganeshmalik_2004@rediffmail.com (G.C.M.); mahua.banerjee@visva-bharati.ac.in (M.B.); 3 International Plant Nutrition Institute, South Asia (East India and Bangladesh) Program, India and African Plant Nutrition Institute, Benguerir 43150, Morocco; s.dutta@apni.net; 4 Department of Biology, College of Science, Taif University, P.O. Box 11099, Taif 21944, Saudi Arabia; es.dessouky@tu.edu.sa (E.S.D.); i.ismail@tu.edu.sa (I.A.I.); 5 National Institute of Oceanography and Fisheries, Kasr ELAiny St. Cairo 101, Egypt; saiftjj@yahoo.com; 6 Department of Botany and Plant Physiology, Faculty of Agrobiology, Food, and Natural Resources, Czech University of Life Sciences Prague, Kamycka 129, 165 00 Prague, Czech Republic; skalicky@af.czu.cz (M.S.); marian.brestic@uniag.sk (M.B.); 7 Department of Plant Physiology, Slovak University of Agriculture, Nitra, Tr. A. Hlinku 2, 949 01 Nitra, Slovakia; 8 Bangladesh Wheat and Maize Research Institute, Dinajpur 5200, Bangladesh

**Keywords:** nutrient management, rice–rice cropping system, growth characters, yield attributes, productivity, nutrient uptake and balance

## Abstract

Rice is the lifeline for more than half of the world population, and in India, in view of its huge demand in the country, farmers adopt a rice–rice cropping system where the irrigation facility is available. As rice is a nutrient-exhausting crop, sustainable productivity of rice–rice cropping system greatly depends on appropriate nutrient management in accordance with the inherent soil fertility. The application of an ample dose of fertilizer is the key factor for maintaining sustainable rice yields and nutrient balance of the soil. Considering the above facts, an experiment was conducted on nutrient management in a rice–rice cropping system at the university farm of Visva-Bharati, situated in a sub-tropical climate under the red and lateritic belt of the western part of West Bengal, India, during two consecutive years (2014–2016). The experiment was laid out in a Randomized Completely Block Design with 12 treatments and three replications, with different rates of N:P:K:Zn:S application in both of the growing seasons, namely, *kharif* and *Boro*. The recommended (ample) dose of nutrients was 80:40:40:25:20 and 120:60:60:25:20 kg ha^−1^ of N:P_2_O_5_:K_2_O:Zn:S in the *Kharif* and *Boro* season, respectively. A high yielding variety, named MTU 7029, and a hybrid, Arize 6444 GOLD, were taken in the *Kharif* and *Boro* seasons, respectively. The results clearly indicated that the application of a recommended dose of nutrients showed its superiority over the control (no fertilizer application) in the expression of growth characters, yield attributes, yields, and nutrient uptake of *Kharif* as well as *Boro* rice. Out of the all treatments, the best result was found in the treatment where the ample dose of nutrients was applied, resulting in maximum grain yield in both the *Kharif* (5.6 t ha^−1^) and *Boro* (6.6 t ha^−1^) season. The corresponding yield attributes for the same treatment in the *Kharif* (panicles m^−2^: 247.9; grains panicle^−1^: 132.0; spikelets panicle^−1^: 149.6; test weight: 23.8 g; and panicle length: 30.6 cm) and *Boro* (panicles m^−2^: 281.6; grains panicle^−1^: 142.7; spikelets panicle^−1^: 157.2; test weight: 24.8 g; and panicle length: 32.8 cm) season explained the maximum yield in this treatment. Further, a reduction or omission of individual nutrients adversely impacted on the above traits and resulted in a negative balance of the respective nutrients. The study concluded that the application of a recommended dose of nutrients was essential for proper nutrient balance and sustainable yields in the rice–rice cropping system.

## 1. Introduction

Rice is one of the most important cereals consumed across the globe and grown in different environmental conditions. A rice–rice cropping system is usually practiced by farmers where sufficient irrigation is available or in favorable lowland rainfed areas [1,2]. Apart from irrigation availability, high consumer demand, a relatively stable market price, and assurance of a minimum support price by the government encourage the farmers to grow two rice crops continuously in consecutive seasons. Though the rice–rice system seems to be feasible from a farmer’s perspective, cereal–cereal cropping systems are often considered unsustainable and are discouraged [3] in terms of nutrient balance in the soil as well as agricultural sustainability [4]. Rice, being a nutrient intensive crop, absorbs a high amount of nutrients. Thus, a rice–rice system is expected to be even more nutrient exhaustive. Unless proper nutrient management practices are followed, soils may develop severe nutrient deficiency over a period of time, negatively affecting agricultural sustainability [5]. Rice–rice cropping systems are most prevalent across a major portion of India as well as South Asia, especially among small and marginal farmers.

Nitrogen (N), phosphorus (P), and potassium (K) are considered as primary nutrients and are very important for the growth and development of rice [6]. Nitrogen is responsible for vegetative growth, improving the leaf area index, chlorophyll synthesis, and so on [7]; thus, increasing photosynthesis and assimilate production in plants. N is deficient in most of the rice-growing areas, which requires a proper focus on nitrogen nutrition [8]. Phosphorus is known for its role in root growth, root development, and reproduction [9]. P is also known to improve tillering and promotes early flowering. Potassium, though not a constituent of organic structures of plants, is very important for plant strength, resistance to biotic and abiotic stresses, and stomatal activity [10]. In addition to primary nutrients, sulphur (S), a secondary plant nutrient, also plays a vital role in plant growth and development as S performs its distinctive role in protein and chlorophyll synthesis [11]. In addition to macronutrients, rice crop also requires micronutrients for completing its life cycle and proper nutrition. Among the different micronutrients, deficiency in zinc (Zn) is commonly observed in rice-growing areas [12], where close to 50% of soils in rice-growing tracts are deficient in Zn [13]. Zinc takes part in the carbohydrate transformation and it is an essential constituent of enzymes such as carbonic anhydrase, superoxide dismutase, and alcohol dehydrogenase [14]. Zinc is also involved in the auxin biosynthesis process. Soil submergence, which is commonly practiced in rice cultivation, results in a deficiency of Zn. Unlike macronutrients, the availability of Zn is higher at a low pH. Alkaline or calcareous soils may result in Zn deficiency [15]. 

A rice–rice cropping system, when practiced, removes nutrients from the same soil depth continuously. If the crops cultivated in a cropping system have a similar nutrient demand and the removal pattern of nutrients from the soil is also the same, then, unless proper care is taken to replenish the nutrient taken up by the crop, a single or multiple nutrient deficiencies may develop over a period of time [16]. Understanding the role of different nutrients in the growth and yield of rice is essential to provide essential nutrients in the required quantity to obtain higher productivity. However, higher productivity should also be sustainable to achieve long-term food security goals.

Imbalanced nutrient application is one of the most important reasons for multi-nutrient deficiency [17]. As the application of nitrogen increases plant dry matter production, a high amount of nitrogen is also expected to increase the uptake of other nutrients. Unless a sufficient amount of other nutrients is applied under such conditions, the crop will continuously drain the native soil nutrients. This, when practiced continuously over years, causes a deficiency in nutrients. Application of nutrients in adequate amounts and suitable proportion is the key to crop nutrition. As the application of all the essential nutrients is practically impossible, those nutrients whose deficiency is prevalent or the nutrients which are yield limiting should be given priority in the nutrient management plan. In addition to those nutrients, nutrients that are expected to be deficient due to huge removal by crops over years in a particular cropping system must be replenished regularly to avoid the development of new nutrient deficiencies. For understanding these phenomena, knowledge regarding the role of important nutrients such as N, P, K, S and Zn in crop growth and yield should be considered. The nutrient balance in the cropping system also should be studied to understand the nutrient removal pattern of the crops under different nutrient combinations. 

The soil and agro-climatic conditions of the red and lateritic belt are unique, and the rice-based cropping system is predominant in the region. The improvement of irrigation facilities and adaptation of HYVs and hybrids attracted farmers to adopt a rice–rice cropping system. As this cropping system is nutrient exhaustive, the development of multi-nutrient deficiency has been observed in recent times [18], drawing the attention of researchers. Similar observations on fertility degradation due to the rice-based cropping system were also noted in Southeast Asia [19]. Under these circumstances, there is an urgent need for balanced nutrient management in intensive cropping systems as a cost-effective and environmentally friendly approach to achieve agricultural sustainability in the region. Taking into consideration the above facts, an experiment was performed to evaluate the impact of different nutrients (inclusive of omission of specific nutrients) management on the growth and productivity of rice in a rice–rice cropping system. The uptake and nutrient balance are also studied to understand the necessity of nutrient supplementation to avoid long-term nutrient deficiencies in a rice–rice cropping system.

## 2. Materials and Methods

### 2.1. Experimental Site 

The site of the field trial was the university farm of Visva Bharati (20°39′ N latitude and 87°42′ E longitude, with an altitude of 58.9 m above M.S.L.), situated in a sub-tropical climate under the red and lateritic belt of the western part of West Bengal, India [20]. The soils of the field trial were sandy loam soil belonging to the typical Ultisols. The characteristics and initial fertility of the experimental soil are described in Table 1.

**Figure 1:**
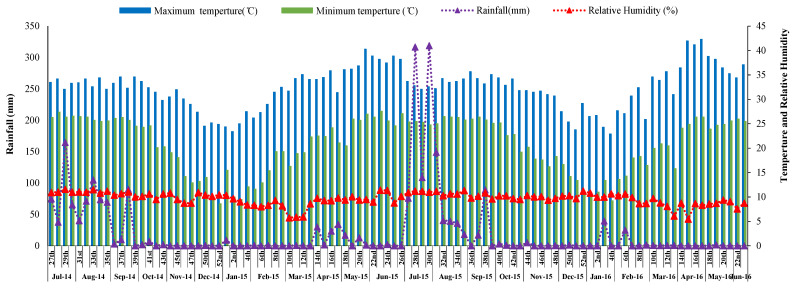
Meteorological data during the crop season (July 2014 to June 2016).

The location falls in the region of the southwest monsoon, and monsoon rains generally start from the end of June and continue up to mid-October, with an average annual rain of 1377 mm. Out of the total annual rain, monsoon rain constitutes about 80–90%. The meteorological information, such as the maximum and minimum temperature (°C), rainfall (mm), and relative humidity (%) during the period of experimentation (July 2014–June 2016), were received from the meteorological observatory of the Institute of Agriculture, Sriniketan, and is presented in Figure 1.

### 2.2. Experimental Design and Treatments 

The experiment on nutrient management in the rice–rice cropping system was carried out for two years (four cropping seasons): 2014–2015 and 2015–2016. The experiment was laid out in a Randomized Complete Block Design (RCBD) with twelve treatments (net plot area of 5 m × 4 m each) and we repeated all treatments three times. The treatments were in the *Kharif* season: T_1_: N_80_P_40_K_40_Zn_25_S_20_; T_2_: N_40_P_40_K_40_Zn_25_S_20_; T_3_: N_0_P_40_K_40_Zn_25_S_20_; T_4_: N_80_P_20_K_40_Zn_25_S_20_; T_5_: N_80_P_0_K4_0_Zn_25_S_20_; T_6_: N_80_P_40_K_20_Zn_25_S_20_; T_7_: N_80_P_40_K_0_Zn_25_S_20_; T_8_: N_80_P_40_K_40_Zn_12.5_S_20_; T_9_: N_80_P_40_K_40_Zn_0_S_20_; T_10_: N_80_P_40_K_40_Zn_25_S_10_; T_11_: N_80_P_40_K_40_Zn_25_S_0_; and T_12_: control, (without any fertilizer); whereas in the *Boro* season: T_1_: N_120_P_60_K_60_Zn_25_S_20_; T_2_: N_60_K_60_Zn_25_S_20_; T_3_: N_0_K_60_Zn_25_S_20_; T_4_: N_120_P_30_K_60_Zn_25_S_20_; T_5_: N_120_P_0_K_60_Zn_25_S_20_; T_6_: N_120_P_60_K_30_Zn_25_S_20_; T_7_: N_120_P_60_K_0_Zn_25_S_20_; T_8_: N_120_P_60_K_60_Zn_12.5_S_20_; T_9_: N_120_P_60_K_60_Zn_0_S_20_; T_10_: N_120_P_60_K_60_Zn_25_S_10_; T_11_: N_120_P_60_K_60_Zn_25_S_0_; and T_12_: control (without any fertilizer). The recommended (ample) dose of nutrients was 80:40:40:25:20 and 120:60:60:25:20 kg ha^−1^ of N:P_2_O_5_:K_2_O:Zn:S in the *Kharif* and *Boro* season rice, respectively, and the treatment T_1_ received an ample dose of nutrients in both the seasons. In the case of T_2_, P, K, S, and Zn were applied in an ample dose, and 50% of the N was applied. In T_3_, N, K, S, and Zn were applied with an ample dose, 0% of N applied, and the same manner was applied for the remaining treatments up to T_11_, but in T_12_, no fertilizer was applied and considered as the control. The total amount of P, K, Zn, and S were applied as basal, while nitrogen was applied in three splits. The HYV and hybrid of rice were taken in the *Kharif* and *Boro* season with the same duration (Table 2). 

### 2.3. Cultural Practices

The standard procedure of rice cultivation in the locality was adopted for both seasons. The treated seeds (with Carbendazim at the rate of 2 g kg^−1^ of seed) were sown in the nursery during both seasons and the seeds were covered lightly with soil. For the main field preparation, the soil was first tilled thoroughly cross wise with a tractor-drawn harrow at an optimum moisture condition. Then tillage was done with a mouldboard plough (25 cm deep) to obtain a good tilth and it was followed by planking. The clods and stubbles of previous crops were removed from the land. The field was flooded with water and the puddling was done under saturated moisture conditions prior to three days of transplanting. After proper levelling, the field was laid out by making net plots (5 m × 4 m each), plot bunds, and channels for irrigation and drainage. After completion of the layout, nutrients were applied as per the treatments. The sources of nitrogen, phosphate, potash, sulphur, and zinc were urea, diammonium phosphate (DAP), muriate of potash (MOP), Bentonite-S (90% of S), and Zn-Ethylenediamine tetraacetic acid (EDTA), respectively. Among the different fertilizers, nitrogen was applied in splits. Half of the nitrogen and a full quantity of the other nutrients were applied as the basal treatment; however, the rest of the N was top-dressed in two equal splits during the maximum tillering and panicle initiation stages for *Kharif* as well as for *Boro* rice.

The 21-day old seedlings were transplanted in the main field at a spacing of 20 cm x 15 cm. In each hill, three seedlings were transplanted. The weeds were removed by hand weeding at early tillering (20 days after transplanting (DAT) and late tillering (40 DAT)). After transplanting, the field was kept saturated with moisture for three weeks to facilitate tillering and followed by a water stagnation of 5 ± 2 cm was maintained up to physiological maturity. Before topdressing of N, standing water was removed from the rice field and irrigated again on the next day and water stagnation was maintained. Ten days prior to harvest, stagnant water was removed. In the *Kharif* season, four irrigations were applied, whereas the in *Boro* season, the crop required six irrigations. The crop faced a mild attack of yellow stem borer and recommended protocols of the university were adopted to manage the pest. However, crop damage due to pest attacks were negligible. The crop was harvested from each net plot manually when it reached 80% maturity. The harvested crop was threshed, winnowed, and the sun-dried weight was recorded at 12% moisture.

### 2.4. Measurements and Analytical Procedures

#### 2.4.1. Growth and Yield Attributes

The third rows from the border of each side of a plot were sampled to record biometric observations. Different growth characters, namely, plant height, dry matter accumulation, leaf area index (LAI), and number of tillers were recorded at different growth stages (20, 40, 60, 80, 100, and 120 days after transplanting, DAT) and the crop growth rate (CGR) was calculated for different periods of the rice–rice cropping system for two consecutive years. For measurement of dry matter accumulation, five randomly selected plants were taken as a destructive sample; the leaves were separated, drying the leaves and the remaining portion of the plant separately in an oven to obtain constant weight (for 48 h at 65 °C). The area of the green leaves taken from the destructive samples was recorded by leaf area meter (Model No: WDY- 500 A, Swastik Scientific Company, India). The ratio of the leaf area weight of these leaves was used to measure the LAI (Equation (1)) [28]. In the case of yield attributes, ten plants from a plot were randomly marked and at crop maturity; these were harvested, dried, and data on the yield parameters were noted.

(1) $$\mathrm{Leaf}\;\mathrm{area}\;\mathrm{index}=\frac{\mathrm{leaf}\;\mathrm{area}\;}{\mathrm{ground}\;\mathrm{area}}\;$$

#### 2.4.2. Collection and Analysis of Plant and Soil Samples

N, P, K, S, and Zn content in plant samples was determined by the standard procedures (Table 1). Plant samples required for the determination of P, K, S and Zn were taken treatment wise after noting down the yields data and dried at 65 °C, pulverized, and digested in di-acid (9:4 *v*/*v*) of nitric acid (HNO_3_)/perchloric acid (HClO_4_). The nutrient content in straw and grain of rice was measured and nutrient uptake was determined by multiplying the nutrient content with the corresponding straw and grain yield [21].

(2) $$\mathrm{Nutrient}\;\mathrm{uptake}\;\left({\mathrm{kg}\;\mathrm{ha}}^{-1}\right)=\frac{\%\;\mathrm{nutrient}\;\mathrm{content}\;\mathrm{in}\;\mathrm{grain}\;\mathrm{or}\;\mathrm{straw}\times \mathrm{dry}\;\mathrm{matter}}{100}$$

Initial soil sample (0–15 cm) was collected prior to cultivation of *Kharif* rice in June 2014 and it was considered for determination of soil characteristics and initial fertility. After each harvest again soil samples were collected treatment wise to obtain the post-harvest soil nutrient status and it was further considered as the initial soil fertility for the next crop. Likewise, the final soil samples were collected in May 2016 after the harvest of *boro* rice. Collected soil samples were air-dried and ground to pass through a 2-mm stainless steel sieve for determination of soil parameters by standard methods as mentioned in Table 1. The initial soil fertility has also been mentioned in Table 1, however, the nutrient balance has been calculated crop-wise as well as for the system.

#### 2.4.3. Nutrient Balance

The balance sheet of available nutrients was computed by using the following formulae given by Tandon [29] (Equation (3)). The determined nutrient balance may be positive or negative.

(3) $$\mathrm{Nutrient}\;\mathrm{balance}\;\left({\mathrm{kg}\;\mathrm{ha}}^{-1}\right)=\mathrm{Available}\;\mathrm{soil}\;\mathrm{nutrient}\;\mathrm{status}-\mathrm{Initial}\;\mathrm{soil}\;\mathrm{status}\;\mathrm{before}\;\mathrm{each}\;\mathrm{crop}$$

### 2.5. Calculations and Statistical Analysis

The experimental data were analysed statistically by using analysis of variance (ANOVA). The standard error of the mean (SEm±) and critical difference at 5% probability level of significance (CD, *p* ≤ 0.05) [30] were calculated. The software used in the statistical analysis and drawing figures (including regression curve) was Excel from Microsoft Office Home and Student version 2019-en-us, Microsoft Inc., Redmond, Washington (DC, USA).

## 3. Results and Discussion

### 3.1. Growth Parameters

Different growth characteristics were calculated for the different periods of the rice–rice cropping system for two consecutive years. The data on plant height (Table 3) revealed that application of an ample dose of nutrients in *Kharif* rice (i.e., T_1_: N_80_P_40_K_40_Zn_25_S_20_) triggered a significant increase in height of the rice plants over the control (i.e., T_12_: no fertilizer) at different days after transplanting (DAT) in both years. The treatment N_80_P_40_K_40_Zn_25_S_20_ (T_1_) produced the tallest rice plants in both years, while the shortest plant was observed in the control plots. However, the treatments T_2_, T_4_, T_6_, T_8_, and T_10_ were statistically on par with the enhancement of plant height in both years. 

In the case of *Boro* rice, the application of T_1_ also produced the longest plants at all growth stages amongst all other treatments during the two years of study (2014–2015 and 2015–2016). The observation clearly showed that the application of 100% recommended dose of N:P:K:Zn:S (also termed as ample dose) increased the plant height at the different growth stages of rice irrespective of seasons, probably because of the proper nutrition obtained by the said treatment. Similar findings were also noted by earlier researchers [31], who also revealed that the application of balanced nutrients in a crop improved the growth and development of plants. 

Dry matter accumulation of the *Kharif* and *Boro* rice in both years (2014 and 2015) was influenced by different levels of nutrients and there was an enhancement in dry matter with the progression of crops towards maturity (Table 4). A strong interrelationship between dry matter accumulation and yield was observed (R_2_ being 0.83 and 0.89, respectively, for the *Kharif* and *Boro* seasons). The treatment T_1_ in both the *Kharif* and *Boro* seasons resulted in the production of the maximum dry matter at all growth stages. The treatment T_1_ in *Kharif* rice increased the dry matter production significantly in both seasons over T_3_, T_5_, T_7_, T_9_, T_11_, and the control, but the treatment T_1_ was statistically on par with T_2_, T_4_, T_6_, T_8_, and T_10_.

A similarity between the two years was noted in *Boro* rice where T_1_ expressed its significant superiority over the control (T_12_, no fertilizer) as observed in different growth stages and T_3_ was statistically on par with control at the harvesting stage during both years of study. The ample dose of nutrients (T_1_) produced significantly more dry matter than T_3_, T_5_, T_7_, T_9_, T_11_, and the control. Although, treatment T_1_ was statistically on par with the T_2_, T_4_, T_6_, and T_10_ treatments in both seasons. The maximum dry matter in the T_1_ treatment was due to the application of 100% of the recommended dose of N:P:K:Zn:S that facilitated access to the required nutrients involving in dry matter production; this assumption was also confirmed by earlier studies [32,33]. 

The data on *Kharif* and *Boro* rice for LAI was measured at different growth stages, where an ample dose of nutrients enhanced the LAI over the control (T_12_, no fertilizer); although, an improvement in the LAI did not differ significantly in all the growth stages in both years (Table 5). The LAI value of rice gradually increased for the *Kharif* and *Boro* rice during both the years for all treatments and reached its maximum values at 60 DAT, followed by a decline as the crops reached maturity. In the case of *Kharif* rice, the maximum LAI was noted at 60 DAT with T_1_ and it was statistically on par with all treatments in 2014 and 2015. Similar to *Kharif* rice, the LAI of *Boro* rice at different growth stages in both years also did not differ significantly for all treatments. Among these treatments, the higher value of LAI was recorded in the T_1_ treatment and the minimum LAI value was in the control plots; although, the LAI for all treatments did not differ significantly. Considering the growth stages, the higher LAI was observed at 60 DAT in both years and the minimum value was at 100 DAT (Table 5). The application of the recommended dose of nutrients produced a higher LAI value, due to the proper nutrition in the plant helping it attaining sufficient vegetative growth (LAI) and keeping the crop healthy irrespective of growth stages and year. This assumption was also confirmed by several earlier studies [34,35], who also revealed an increase in LAI with the recommended dose of N:P:K:Zn:S.

The number of tillers m^−2^ of *Kharif* and *Boro* rice was also influenced by nutrient management, observed during two consecutive years (Table 6). Application of an ample dose of nutrients, i.e., N_80_P_40_K_40_Zn_25_S_20_ in *Kharif* rice and N_120_P_60_K_60_Zn_25_S_20_ in *Boro* rice, resulted in the production of the maximum number of tillers over the control at all the growth stages. Considering the growth stages, tillers m^−2^ of the *Kharif* rice at 20 DAT were significant in both years, and 40 and 60 DAT only for all treatments; although, the maximum tillers m^−2^ was recorded in T_1_ and the lowest was in the T_12_ (control) treatment. With little exception, tillers m^−2^ for T_1_ was statistically on par with T_2_, T_4_, T_6_, T_8_, and T_10_ in increasing the number of tillers during both years; however, T_1_ significantly produced more tillers than T_3_, T_5_, T_7_, T9, T_11_, and T_12_ (control). 

A similar trend was also noted in *Boro* rice, where an ample dose of recommended nutrients (T_1_) produced maximum tillers per unit area. Treatment T_1_ showed its significant superiority to T_3_, T_5_, T_7_, T_9_, T_11_, and the control (T_12_, no fertilizer) during both years, but T_1_ remained statistically on par with T_2_, T_4_, T_6_, T_8_, and T_10_. Omission of all nutrients in T_12_ was totally dependent on inherent soil fertility and, due to lack of sufficient nutrients, it did not produce the desired number of tillers. On the other hand, the treatment T_1_ received an ample dose of recommended nutrients that facilitated proper nutrition and resulted in maximum tillers per unit area at different growth stages of *Kharif* and *Boro* rice during both the years of study. The beneficial effects of fertilizers in enhancing tillers were earlier observed by researchers [36,37].

### 3.2. Yield Attributes and Yield

Yield attributes such as panicles m^−2^, grains panicle^−1^, spikelets panicle^−1^, test weight, and panicle length were recorded for both *Kharif* and *Boro* rice during both seasons (Table 7). Considering both seasons data of these parameters, panicles m^−2^, grains panicle^−1^, spikelets panicle^−1^, and test weight varied significantly only in the first season. The recommended dose of nutrients (T_1_) registered higher values than T_3_, T_5_, T_7_, T_9_, T_11_, and T_12_ (control) in both years. However, the treatment with an ample dose of nutrients (T_1_) was statistically on par in increasing the values of the yield attributes.

The yield-attributing characters of *Boro* rice did not differ significantly in both years, where T_1_ exerted higher values over T_3_, T_5_, T_7_, T_9_, T_11_, and the control (T_12_, no fertilizer) during 2014–2015 and 2015–2016. However, T_1_ was statistically on par with the T_2_, T_4_, T_6_, T_8_, and T_10_ treatments. The results corroborate the findings of earlier studies [34,35], where researchers revealed that balanced doses of all nutrients influence the proper growth and development of plants, leading to improved yield-attributing characters of rice. 

**Figure 2:**
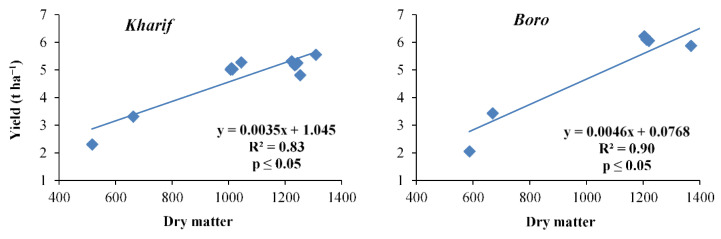
Relation between dry matter and yield of *Kharif* and *Boro* rice.

**Figure 3:**
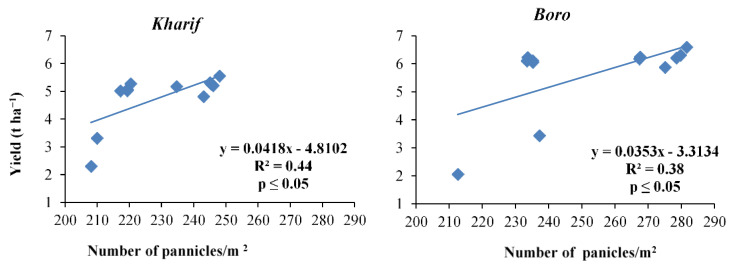
Relation between number of panicles m^−^^2^ and yield of *Kharif* and *Boro* rice.

**Figure 4:**
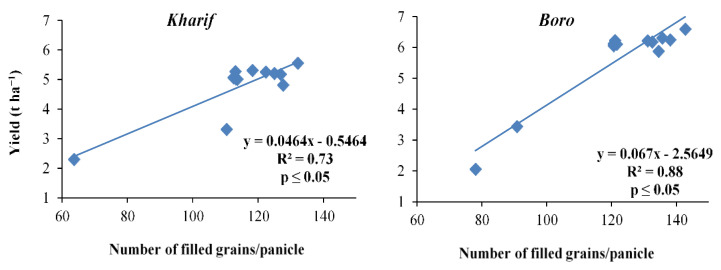
Relation between number of filled grains panicle^−^^1^ and yield of *Kharif* and *Boro* rice.

**Figure 5:**
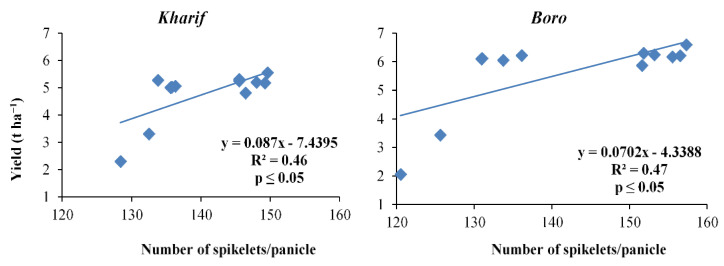
Relation between number of spikelets panicle^−^^1^ and yield of *Kharif* and *Boro* rice.

**Figure 6:**
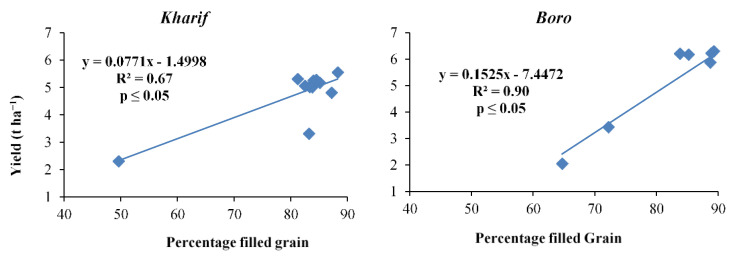
Relation between percentages filled grain and yield of *Kharif* and *Boro* rice.

**Figure 7:**
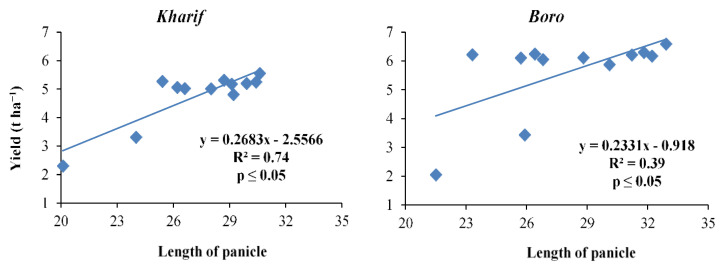
Relation between length of panicle and yield of *Kharif* and *Boro* rice.

**Figure 8:**
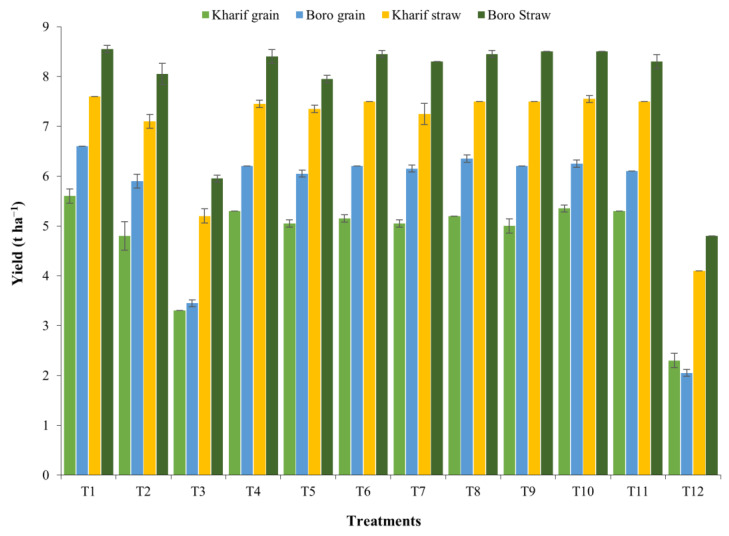
Effect of nutrient management on the yield of *Kharif* rice. SE± in each bar was calculated from three replications for every treatment.

The ‘R’ values of the yield attributes were reflected in the productivity of the *Kharif* and *Boro* rice in terms of grain and straw yields during both the years of study (Figure 2, Figure 3, Figure 4, Figure 5, Figure 6 and Figure 7). The data showed that the grain yield of *Kharif* rice was at its maximum (5.46 and 5.67 t ha^−1^ in 2014 and 2015, respectively) with the treatment T_1_ (Figure 8). In 2014, the treatment with an ample dose of nutrients (T_1_) produced significantly more grain yield than T_2_, T_3_, and the control (T_12_); but, in 2015, T_1_ yielded significantly more than T_3_ and the control. The other treatments were statistically on par with T_1_. The grain yield of *Boro* rice was higher with the application of an ample dose of the recommended fertilizer (T_1_) that yielded 6.6 t ha^−1^ in both years. The treatments were statistically on par with the other treatments, except for T_2_, T_3_, and T_12_ (unfertilized control), in increasing the grain yield of *Boro* rice during both years. In the rice–rice cropping system also, both the *Kharif* and *Boro* rice yielded more with the ample dose of nutrients application. A similar type of impact of an ample dose of recommended nutrients was earlier noted by Mohapatra [38] and Trivedi et al. [39].

Nutrient levels influenced straw yield in the rice–rice cropping system as noted in the case of grain yield. The treatment comprising recommended dose of N+P+K+S+Zn resulted in significant improvement in straw yield of *Kharif* and *Boro* rice over unfertilized control treatments during both the years of experimentation (Figure 8). In *Kharif* rice, the treatment T_1_ produced the maximum straw yield and it was significantly more than T_3_ and T_12_ (unfertilized control) in 2014, but in 2015, the application of T_1_, being statistically on par with the other nutrient management treatments, significantly registered more straw yield over the control (T_12_). The straw yield of *Boro* rice was maximum with T_1_ and it remained significantly more than T_3_ and T_12_ (control) during both 2014–2015 and 2015–2016. As the ample dose of nutrient application produced more dry matter than the control, grain and straw yields also followed a similar trend because the maximum biomass production was reflected with said treatment. The results conform with the findings of Trivedi et al. [39], who also noted higher biomass production with the recommended dose of nutrients in rice.

### 3.3. Nutrient Uptake

The uptake of N, P, K, S, and Zn by the grain and straw of *Kharif* and *Boro* rice were obtained by multiplying the grain and straw yield with the nutrient content of the grain and straw of the respective treatments. The results are presented below (Table 8, Table 9 and Table 10).

#### 3.3.1. Nitrogen Uptake

In 2014, the highest nitrogen uptake in *Kharif* season rice grain (68.5 kg ha^−1^) was with the treatment T_1_, which was significantly higher than T_2_, T_3_, T_4_, T_5_, T_8_, and the control (T_12_); however, T_1_ remained statistically on par with T_6_, T_7_, T_9_, T_10_, and T_11_ (Table 8). In 2015, T_1_ also resulted in the maximum N uptake by grain and, it being statistically on par with T_4,_ T_6_, T_8_, T_10_, and T_11_, was significantly superior to T_2_, T_3_, T_5_, T_7_, T_9_, and T_12_ (control). Similarly, the nitrogen uptake by straw was also maximum with an ample dose of fertilizer application. In the case of nitrogen uptake by rice straw, T_1_ removed the maximum nitrogen and it remained statistically on par with T_4_, T_5_, T_6_, T_7_, T_8_, T_9_, T_10_, and T_11_ in 2014 and with T_5_, T_7_, T_8_, T_9_, T_10_, and T_11_ in 2015. Interestingly, T_1_ remained on par with those treatments that received 80 kg N ha^−1^ in the *Kharif* season. The least quantity of N was removed by the rice straw with the unfertilized control (T_12_) during both years of experimentation. Greater values of N uptake by the grain and straw were noted with the recommended dose of N fertilizer application for *Kharif* rice (80 kg ha^−1^) during both the years, and it was probably the proper utilization of applied N fertilizer by crops into biomass (grain and straw yields) production.

During the *Boro* seasons of 2014–2015 and 2015–2016, the highest nitrogen uptake by rice grain (98.8 and 107.6 kg ha^−1^) was recorded with the treatment T_1_ and the least quantity was noted with the unfertilized control (T_12_) (Table 9). The result of N uptake by grain in 2014–2015 revealed that 100% RDF (T_1_) removed the maximum nitrogen from the soil and it was significantly more than T_2_, T_3_, T_5_, and T_12_ (control). The remaining treatments were statistically on par with T_1_ in the expression of nutrient uptake by grains of *Boro* rice in 2014–2015. However, in the case of 2015–2016, T_1_ registered more nitrogen uptake by grains of *Boro* rice, which was further significantly more than T_2_, T_3_, T_7_, T_11_, and T_12_ (control). N uptake by *Boro* rice grains was drastically reduced in T_3_, which was statistically on par with the unfertilized control (T_12_) during both years of experimentation. Rice is basically a nutrient-draining crop and in the rice-rice cropping system, the second crop (*Boro* rice) did not get any nitrogen in T_3_, probably due to insufficient supply of the primary nutrient (N); thus, the treatment performed poorly.

In 2014–2015, *Boro* rice straw registered its maximum uptake of nitrogen at T_1_ and it was statistically on par with T_5_, T_6_, and T_7_. T_12_ (unfertilized control) expressed the least value and it was statistically on par with T_3_. Both T_3_ and T_12_ (control) were significantly inferior to other treatments in 2014–2015 in N uptake by straw. However, in 2015–2016, T_1_ was statistically on par with all other treatments except T_3_ and T_12_ (control). The variation in N uptake during two consecutive years among treatments was probably due to variation in yields. In both years, T_3_ and T_12_ (control) performed poor in nitrogen uptake by straw because nitrogenous fertilizer was not applied in these treatments. Earlier researchers evidenced that an ample dose of fertilizer application recorded more uptake of nitrogen by grains and straws of rice [40,41].

#### 3.3.2. Phosphorus Uptake

In 2014, the uptake pattern of P was influenced by the yield of *Kharif* rice grain and straw (Table 9). The highest P uptake in rice grain (19.30 kg ha^−1^) in 2014 was noted with the treatment T_1_, which was statistically on par with T_6_, T_7_, T_8_, T_9_, T_10_, and T_11_. The grains of *Kharif* rice in 2015 also removed the maximum P by T_1_; however, it was statistically on par with only T_6_ and T_10_. The lowest quantity of P uptake by the *Kharif* rice grains was recorded with T_12_ (control) during both years. A similar trend in P uptake by rice straw during the *Kharif* season was noted in 2014 and 2015 where the treatment T_1_ resulted in their maximum values (11.8 and 14.1 kg ha^−1^, respectively). In 2014, T_1_ registered significantly more nutrient uptake by rice straw during the *Kharif* season than T_3_, T_4_, T_5_, and the control (T_12_); but T_1_ was also statistically on par with T_2_, T_6_, T_7_, T_8_, T_9_, T_10_, and T_11_. The treatment (N_80_P_20_K_40_Zn_25_S_20_) also removed the maximum P by straw in *Kharif* of 2015, and the treatment is statistically on par with T_2_, T_7_, T_8_, T_9_, T_10_, and T_11_, but significantly superior to T_3_, T_4_, T_5_, T_6_, and the control.

In the case of *Boro* rice, a similar trend was observed in terms of nutrient uptake by rice grain and straw during both the years (2014–2015 and 2015–2016) (Table 10). An ample dose of recommended fertilizer application (T_1_) registered significantly more P uptake during both the years by *Boro* rice grains over T_2_, T_3_, T_4_, T_5_, and T_12_ (unfertilized control) and the treatment with the application of T_1_ remained statistically on par with T_6_, T_7_, T_8_, T_9_, T_10_, and T_11_. Similarly, straw of *Boro* rice showed maximum P uptake by T_1_ and the treatment being statistically on par with T_6_, T_7_, T_8_, T_9_, T_10_, and T_11_ resulted in significantly greater P uptake by the straw of *Boro* rice than T_3_, T_4_, T_5_, and T_12_ (control). Research evidence proved that an ample dose of the recommended dose of nutrient application showed greater uptake of P by grains of rice [42,43].

#### 3.3.3. Potassium Uptake

Potassium uptake by grains of *Kharif* rice was maximum at an ample dose of recommended fertilizer application (T_1_) during both years of experimentation (Table 9). The treatment T_1_, being statistically on par with T_2_, T_4_, T_5_, T_8_, T_9_, T_10_, and T_11_, registered its significant superiority to T_3_, T_6_, T_7_, and unfertilized control (T_12_) in the *Kharif* season of 2014 for potassium removal by grains. However, in 2015, only a few treatments, namely T_2_, T_9_, T_10_, and T_11_, remained statistically on par with T_1_ in increasing the K uptake by *Kharif* rice grains and the treatment T_1_ recorded significantly more uptake of said primary nutrients over the remaining treatments. Further, it was also noted that the treatment T_12_ recorded the least quantity of K during both the years and, in *Kharif* 2015, the rice grains registered a comparatively less amount of K removal because of the non-application of nutrients in the two consecutive years. A similar trend was also noted in K uptake by the straw of *Kharif* rice during both the years as the treatment T_1_ showed the maximum uptake. In both the years, straw of *Kharif* rice removed the maximum amount of K with the treatment T_1_ and it was statistically on par with T_2_, T_4_, T_5_, T_8_, T_9_, and T_10_. In 2014, T_1_ remained significantly superior to T_11_, but in 2015 both the treatments were statistically on par in the removal of K by *Kharif* rice straw.

The data on potassium uptake (kg ha^−1^) showed that during the *Boro* season (Table 10), the uptake of K in grain was the highest with T_1_ and it was statistically on par with T_2_, T_4_, T_5_, T_8_, T_9_, T_10_, and T_11_ in 2014–2015_._ The lowest value was recorded in T_12_ (control) and it was significantly inferior to all other treatments. Interestingly, in 2015–2016, K uptake by the rice grains was less than the previous year with the same treatment (T_12_) and that clearly indicated the continuous removal of stored nutrients because in the treatment no nutrients were added in the consecutive two years. In the *Boro* season of 2015–2016, an ample dose of nutrients application (T_1_) registered the maximum K uptake by rice grains and the treatment was statistically on par with T_4_, T_5_, T_8_, T_9_, and T_11_. Like other major nutrients, K uptake of rice straw during the *Boro* season of 2014–2015 was noted as at a maximum at T_1_ and, it being statistically on par with T_2_, T_4_, T_5_, T_8_, T_9_, and T_10_, remained significantly superior to T_3_, T_5_, T_6_, T_9_, and the unfertilized control (T_12_) in 2015–2016. However, in 2015–2016, the treatment T_1_ was observed to remove significantly more K by *Boro* rice straw than T_3_, T_5_, T_6_, T_7_, T_10_, T_11_, and T_12_ (control). The results noted that higher removal of K by rice with the recommended dose of fertilizer application [44,45].

#### 3.3.4. Zinc Uptake

Zn uptake (kg ha^−1^) by *Kharif* rice grain and straw was influenced by nutrient management treatments during two consecutive years of study (Table 9). In 2014, rice grains registered their maximum Zn uptake by T_1_, and it remained statistically on par with T_2_, T_4_, and T_6_ and significantly superior to the rest of the treatments. In 2015, T_1_ remained statistically on par with T_2_ and T_10_ in the uptake of Zn by rice grains; however, T_1_ recorded significantly more Zn uptake over other treatments. Similarly, rice straw also recorded its maximum Zn uptake with T_1_ during the *Kharif* seasons of 2014 and 2015. In 2014, T_1_ was statistically on par with T_4_, T_6_, and T_11_; but, in 2015, treatment T_1_, being statistically on par with T_6_ and T_7_, recorded significantly more Zn uptake by the straw of *Kharif* rice over other treatments. Moreover, the control treatment (T_12_) recorded the lowest uptake of Zn during both the years by grain and straw of *Kharif* rice because of it being devoid of any fertilizer application.

*Boro* rice grain and straw also showed a similar trend in Zn uptake as the maximum uptake of the micronutrient was noted with an ample dose of recommended fertilizer application (T_1_) in both years (Table 10). An ample dose of the recommended fertilizer (T_1_) was statistically on par with T_2_, T_6_, T_7_, T_10_, and T_11_ in Zn uptake by *Boro* rice grains; however, the treatment was significantly superior to the remaining treatments in 2014–2015 and 2015–2016. Similarly, the *Boro* rice straw removed the maximum Zn with the treatment T_1_ and it is statistically on par with T_2_, T_7_, T_10_, and T_11_, registering more Zn uptake than other treatments during both years. As noted in the other treatments, the least quantity of Zn uptake by grain and straw of *Boro* rice was recorded with the unfertilized control treatment (T_12_). The results are in agreement with the research evidence of Mohapatra [38], Pampolinoa et al. [46], and Chandrapala et al. [47], who earlier noted a higher quantity of Zn removal by rice with an ample dose of Zn-containing fertilizers.

#### 3.3.5. Sulphur Uptake

In the rice–rice cropping system, S uptake was influenced by nutrient management in the *Kharif* season (Table 9). During both the years (2014 and 2015), *Kharif* rice grains registered their maximum S uptake by the treatment T_1_ and, it being statistically on par with T_2_, T_4_, T_6_, T_7_, T_8_, and T_9_, recorded more S uptake over the remaining treatments. In the case of rice straw, in 2014, T_1_ was statistically on par with T_2_, T_7_, and T_8_; and in 2015, the treatment T_1_, being statistically on par with T_2_, T_4_, T_6_, T_7_, T_8_, and T_9_, registered significantly more S uptake than the other treatments during the *Kharif* season. As expected, the control treatment (T_12_, control) recorded the least S uptake by the *Kharif* rice grain and straw and it was significantly inferior to all other treatments during both the years under study.

A similarity was noted in S uptake by *Boro* rice grain and straw during both years (Table 10). The treatment T_1_ recorded the maximum S uptake by *Boro* rice grain and straw and it was statistically on par with T_2_, T_4_, T_6_, T_7_, and T_8_ during both years. However, T_1_ was significantly superior to the other treatments in S removal by the grain and straw of *Boro* rice in 2014–2015 and 2015–2016. The treatment with no fertilizer (T_12_, control) showed significant inferiority over other treatments for S removal by grain and straw of *Boro* rice during both the years under study. The results clearly indicated that application of S was required in the rice–rice cropping system for proper nutrition of the crops. The results conform with the findings of Porpavai et al. [48] and Singh et al [49].

### 3.4. Nutrient Balance

The initial nutrient status of the soils before transplanting of *Kharif* rice was analysed and recorded (Table 10). The nutrients were added through chemical fertilizers as per the treatments for rice crops in the rice–rice cropping system. The removal of nutrients by the rice crop was quantified after the harvest of each crop during the *Kharif* and *Boro* season in two consecutive years. The nutrient balance was measured after the final harvest of *Boro* rice in 2015–2016. The rice–rice cropping system removed a considerable amount of nutrients during the two years of study and the ample dose of recommended fertilizer application recorded the maximum quantity of nutrient (N, P, K, Zn, and S were considered in the experiment) removal. As expected, the control treatment (no fertilizer application) yielded less with the least nutrient uptake. After completion of two years of the experiment, it was observed that omission of any nutrient, as well as a control treatment, resulted in a negative nutrient balance, which is synonymous with depletion of soil fertility. The results clearly showed that to achieve crop yields on a sustainable basis one would need to apply the recommended fertilizers, and these recommendations should be made based on crop demand (removal) and the inherent soil nutrient-supplying capability. A similar type of observation was earlier noted by researchers [50,51].

As the rice–rice cropping system is the most prevalent system for irrigated lands of the red and lateritic belt of West Bengal, the nutrient balance must be kept into consideration for agricultural sustainability. Further, being a nutrient draining system, rice–rice systems remove a sizable quantity of nutrients, causing multi-nutrient deficiency problems—a threat to sustainable farm output—which is unlike other rice-based cropping systems, such as rice–legume systems, which has the opportunity to replenish a portion of the nutrients (more specifically N) through biological N fixation and nutrient recycling. In the rice–rice cropping system, the soil remains flooded for a long period and in this condition, loss of N and non-availability of Zn further aggravate the dimension to improper plant nutrition. Under rice–rice cropping systems, exogenous application of nutrients is vital for nutrient supply to crops. The experimental results of the present study also revealed that the application of ample doses of recommended nutrients is essential to maintain a positive nutrient balance.

## 4. Conclusions

Without proper and balanced nutrient management practices, the rice-rice system can prove to be highly unsustainable and can drain the soil nutrients quickly. Hence, understanding the nutrient requirement, nutrient removal, and nutrient balance of this system is essential. Nitrogen, phosphorus, potassium, zinc, and sulphur are the nutrients of topmost priority, as their deficiency is widespread. These nutrients also play a crucial role in deciding crop performance. In the experiment, a rice–rice cropping system was studied concerning different nutrient management options. The imbalanced or insufficient nutrient application affects crop nutrient removal, thus affecting the growth and development of the plant. In addition to this, inappropriate nutrient supply over a long period reduces soil fertility, especially when a nutrient-exhausting cropping system such as a rice–rice cropping system is practised. The treatment where ample nutrients were provided proved to be most effective in improving the growth parameters, yield-attributing characteristics, and yield of rice in both the *Kharif* and *Boro* seasons. Ample nutrient application also helped to replenish the nutrients removed by the rice–rice cropping system. Imbalanced and insufficient nutrient application may make a nutrient-intensive cropping system, such as a rice–rice cropping system, unsustainable and low yielding. Considering this, an ample dose of nutrients in balanced proportions may be recommended to farmers of eastern India to maintain both productivity and agricultural sustainability and also to avoid long-term nutrient deficiencies in the rice–rice cropping systems of the region. Balanced nutrient management in cropping systems, thereby minimizing environmental pollution, is a cost-effective and environmentally friendly approach to target agricultural sustainability.

## Figures and Tables

**Table 1:** Characteristics and initial fertility of the experimental soil and methodologies followed for determination of soil quality.

Particulars	Characters/Value	Status	Methodology	References
Texture	Sandy loam	-	Hydrometer method	[21]
pH	5.65	Acidic	Determined by pH meter in 1:2.5 ratio of soil–water suspension	[22]
Electrical conductivity (EC) (dS m^−1^)	0.26	-	Solubridge method	[22]
Organic carbon (%)	0.35	Low	Walkley and Black method	[22]
Available nitrogen (kg ha^−1^)	230.0	Low	Alkaline permanganate method	[23]
Available phosphorous (kg ha^−1^)	11.2	Low	Bray’s method	[24]
Available potassium (kg ha^−1^)	125.2	Medium	Flame photometer method	[25]
Zinc (mg kg^−1^)	0.22	Low	Diethylenetriaminepentaacetate (DTPA) extractable Zn determination by Atomic Absorption spectroscopy (AAS)	[26]
Sulphur (kg ha^−1^)	10.5	Low	Turbidimetric Method	[27]

**Table 2:** Variety/hybrid chosen, date of transplanting, and duration of rice (2014–2016).

Particulars	*Kharif*	*Boro*
Cropping system	Rice	Rice
Variety/hybrid	HYV (High yielding variety) rice variety: MTU 7029	Hybrid rice: Arize 6444 GOLD
Date of transplanting	3 August 2014; 2 August 2015	2 February 2015 and 2016
Duration	150 days	150 days

**Table 3:** Effect of nutrient management on the plant height (cm) of *Kharif* and *Boro* rice at different growth stages.

Treatments	Plant Height (cm)											
	20 DAT		40 DAT		60 DAT		80 DAT		100 DAT		120 DAT	
	2014	2015	2014	2015	2014	2015	2014	2015	2014	2015	2014	2015
***Kharif* rice**												
T_1_	46.3 a	49.9 a	61.7 a	64.8 a	98.0 a	99.5 a	118.0 a	119.4 a	118.9 a	119.6 a	119.7 a	119.5 a
T_2_	41.9 ab	46.2 a	59.2 ab	62.4 a	84.9 abc	91.4 ab	108.9 a	111.8 a	110.0 abc	111.9 abc	110.2 a	112.0 a–e
T_3_	29.2 cd	26.1 b	40.0 cd	38.4 e	74.5 cd	70.0 c	94.5 a	92.9 a	96.1 bc	93.8 bc	96.8 a	94.0 de
T_4_	44.1 a	44.8 a	60.1 ab	60.1 ab	95.0 ab	95.9 ab	115.0 a	116.1 a	116.6 a	116.6 ab	116.8 a	117.2 ab
T_5_	38.7 ab	39.4 ab	48.3 bcd	49.2 d	79.9 bcd	80.9 ac	95.9 a	97.6 a	96.2 c	98.6 abc	98.5 a	98.9 cde
T_6_	45.3 a	45.4 a	57.9 ab	60.1 ab	94.3 ab	93.6 ab	113.3 a	114.1 a	114.5 ab	114.4 ab	114.7 a	115.4 ab
T_7_	41.6 ab	40.2 a	51.8 abc	52.0 bcd	83.0 a–d	81.2 bc	96.3 a	90.8 a	96.4 bc	91.3 bc	96.6 a	92.5 b–e
T_8_	43.8 ab	44.4 a	57.1 ab	59.0 abc	91.9 ab	94.7 ab	111.9 a	113.9 a	111.8 abc	114.7 ab	111.7 a	116.6 abc
T_9_	34.8 bcd	34.0 ab	50.8 a–d	51.2 bcd	82.5 a–d	81.1 bc	97.1 a	94.3 a	98.0 bc	95.5 abc	98.9 a	96.7 a–e
T_10_	41.2 ab	45.6 a	59.2 ab	57.0 a–d	85.0 abc	95.0 ab	115.0 a	110.7 a	116.2 a	112.2 abc	116.3 a	112.1 a–d
T_11_	37.4 abc	38.7 ab	51.9 abc	50.2 cd	80.7 acd	80.0 bc	98.7 a	93.0 a	98.1 bc	94.6 bc	98.1 a	93.8 a–e
T_12_	26.1 d	25.6 b	38.3 d	34.5 e	68.3 d	67.0 c	88.3 b	86.3 b	88.5 c	87.5 c	88.6 a	87.6 e
F-test	**	**	**	**	**	**	*	*	**	**	*	**
SEm (±)	2.5	3.0	3.3	4.2	5.1	6.0	6.5	6.2	6.3	6.3	6.6	6.3
CV (%)	7.12	13.7	8.12	12.3	6.56	7.17	10.0	11.4	8.43	8.18	9.96	8.21
***Boro* rice**												
T_1_	51.3 a	52.9 a	82.0 a	84.2 a	104.1 a	106.2 a	121.0 a	124.1 a	122.0 a	124.0 a	122.0 a	124.8 a
T_2_	46.9 a	48.6 ab	72.3 abc	76.4 abc	103.8 a	104.7 a	116.2 ab	117.4 abc	116.0 ab	118.0 abc	117.0 a	119.0 a
T_3_	34.1 bc	33.1 c	56.3 de	52.6 e	78.3 b	74.4 b	97.4 a–e	94.6 bcd	98.4 abc	94.3 cd	98.4 ab	94.4 ab
T_4_	49.1 a	50.1 ab	74.3 ab	84.9 a	103.6 a	104.4 a	118.9 a	116.1 abc	118.0 ab	117.0 abc	118.0 a	116.8 ab
T_5_	44.6 abc	45.2 ab	68.5 bcd	68.3 cd	80.0 b	78.8 b	91.9 cde	93.0 cd	95.4 bc	94.2 cd	95.2 ab	94.5 ab
T_6_	47.1 a	47.9 ab	75.1 ab	81.2 a	102.9 a	103.7 a	117.1 ab	117.6 abc	119.0 ab	119.0 ab	120.0 a	118.9 a
T_7_	45.1 abc	44.2 abc	69.1 bc	69.0 bcd	84.4 ab	85.4 ab	89.9 de	97.1 bcd	99.1 abc	97.6 bcd	99.3 ab	98.1 ab
T_8_	48.8 a	49.4 ab	70.3 abc	78.7 ab	103.1 a	104.3 a	115.1 abc	123.3 a	118.0 ab	125.0 a	118.0 a	124.7 a
T_9_	41.8 abc	42.4 abc	70.0 abc	68.1 cd	86.4 ab	78.3 b	91.5 cde	98.2 bcd	101.0 abc	98.6 abc	101.2 ab	98.9 ab
T_10_	46.2 ab	47.9 ab	75.3 ab	83.7 a	104.5 a	105.3 a	112.3 a–d	119.4 ab	116.0 ab	119.0 ab	115.0 ab	119.2 a
T_11_	40.2 abc	40.8 bc	60.7 cde	64.0 d	86.9 ab	87.1 ab	93.4 b–e	94.5 cd	100.0 abc	97.9 bcd	100.1 ab	98.2 ab
T_12_	33.1 c	32.3 c	54.0 e	51.0 e	71.9 b	69.7 b	83.0 e	80.2 d	86.5 c	81.6 d	87.0 b	81.7 b
F-test	**	**	**	**	**	**	**	**	**	**	**	**
SEm (±)	1.8	2.0	4.1	5.0	5.6	6.4	7.5	7.5	6.5	8.8	6.8	7.5
CV (%)	9.26	9.14	6.26	4.83	7.50	7.77	7.77	7.85	8.09	7.62	9.27	11.5

***Kharif* season:** T_1_: N_80_P_40_K_40_Zn_25_S_20_; T_2_: N_40_P_40_K_40_Zn_25_S_20_; T_3_: N_0_P_40_K_40_Zn_25_S_20_; T_4_: N_80_P_20_K_40_Zn_25_S_20_;T_5_: N_80_P_0_K4_0_Zn_25_S_20_; T_6_: N_80_P_40_K_20_Zn_25_S_20_; T_7_: N_80_P_40_K_0_Zn_25_S_20_; T_8_: N_80_P_40_K_40_Zn_12.5_S_20_;T_9_: N_80_P_40_K_40_Zn_0_S_20_; T_10_: N_80_P_40_K_40_Zn_25_S_10_; T_11_: N_80_P_40_K_40_Zn_25_S_0_ and T_12_: control; (without any fertilizer); ***Boro* season:** T_1_: N_120_P_60_K_60_Zn_25_S_20_; T_2_: N_60_K_60_Zn_25_S_20_; T_3_: N_0_K_60_Zn_25_S_20_; T_4_: N_120_P_30_K_60_Zn_25_S_20_; T_5_: N_120_P_0_K_60_Zn_25_S_20_; T_6_: N_120_P_60_K_30_Zn_25_S_20_; T_7_: N_120_P_60_K_0_Zn_25_S_20_; T_8_: N_120_P_60_K_60_Zn_12.5_S_20_; T_9_: N_120_P_60_K_60_Zn_0_S_20_; T_10_: N_120_P_60_K_60_Zn_25_S_10_; T_11_: N_120_P_60_K_60_Zn_25_S_0_ and T_12_: control (without any fertilizer). CV (%) = coefficient of variation; ** and * significant at *p* ≤ 0.01 and *p* ≤ 0.05, respectively; NS = not significant; different letters within the continuous columns indicate significant differences at the 1% level of probability.

**Table 4:** Effect of nutrient management on dry matter accumulation (g) of *Kharif* and *Boro* rice at different growth stages.

Treatment	Dry Matter Accumulation (g m^−2^)											
	20 DAT		40 DAT		60 DAT		80 DAT		100 DAT		120 DAT	
	2014	2015	2014	2015	2014	2015	2014	2015	2014	2015	2014	2015
***Kharif* rice**												
T_1_	156.1 a	158.2 a	416.0 a	420.9 a	760.3 a	768.0 a	1170.0 a	1181.5 a	1280.0 a	1290.9 a	1301.1 a	1315.5 a
T_2_	150.8 a	153.0 a	390.1 ab	409.5 a	728.9 ab	750.3 a	1134.4 abc	1137.6 a	1240.6 a	1243.8 a	1247.6 a	1257.8 a
T_3_	140.8 a	140.3 a	305.9 bc	302.3 ab	510.1 cd	518.9 bc	602.3 d	606.9 c	657.6 b	658.3 d	660.6 bc	662.8 d
T_4_	153.6 a	153.8 a	404.0 a	407.3 a	735.4 ab	750.5 a	1132.1 abc	1138.7 a	1225.9 a	1236.6 a	1235.9 a	1249.2 ab
T_5_	141.0 a	140.8 a	370.4 ab	373.8 ab	506.7 cd	507.8 bc	900.0 c	906.3 b	1002.9 a	1006.9 c	1009.9 ab	1016.4 c
T_6_	155.2 a	157.3 a	407.3 a	409.9 a	745.6 ab	750.7 a	1140.9 ab	1140.0 a	1222.8 a	1233.0 a	1230.8 a	1235.6 ab
T_7_	140.7 a	142.9 a	373.9 ab	357.3 ab	521.9 cd	505.2 bc	903.3 c	902.2 b	1000.4 a	1008.8 c	1006.4 ab	1012.4 c
T_8_	155.0 a	159.2 a	406.9 a	410.3 a	742.9 ab	750.5 a	1110.8 abc	1137.6 a	1209.8 a	1225.6 a	1229.8 a	1245.6 ab
T_9_	140.4 a	147.6 a	374.2 ab	380.6 ab	506.6 cd	508.3 bc	909.8 bc	908.2 b	1002.1 a	1005.3 c	1003.1 ab	1009.7 c
T_10_	155.7 a	156.5 a	403.5 a	404.8 a	745.5 ab	749.5 a	1108.3 abc	1111.7 a	1210.1 a	1215.9 ab	1220.1 a	1225.5 ab
T_11_	140.0 a	147.1 a	363.0 ab	370.5 ab	590.0 bc	609.7 ab	950.3 abc	952.7 b	1040.3 a	1040.5 bc	1042.3 a	1045.7 bc
T_12_	101.7 b	100.5 b	260.0 c	255.3 b	408.8 d	407.5 c	508.3 d	505.3 c	535.2 b	532.3 d	518.0 c	514.5 da
F-test	*	*	**	**	**	**	**	**	*	**	**	**
SEm (±)	5.1	3.6	13.7	12.6	33.4	26.4	49.7	47.4	52.3	61.5	57.0	72.3
CV (%)	7.21	5.92	7.96	13.29	8.86	10.04	8.25	4.63	10.30	5.86	11.74	6.43
***Boro* rice**												
T_1_	177.1 a	180.0 a	437.0 a	452.5 a	870.3 a	885.7 a	1370.0 a	1380.7 a	1507.2 a	1511.1 a	1519.3 a	1515.1 a
T_2_	175.8 ab	175.4 a	411.1 a	415.8 ab	830.9 a	831.6 a	1300.4 a	1307.0 a	1457.7 a	1408.6 a	1424.7 a	1410.6 a
T_3_	155.8 a	154.5 ab	308.9 a	304.6 c	421.1 b	410.5 b	656.3 b	652.6 b	758.4 b	768.6 b	759.2 b	766.7 b
T_4_	172.6 ab	168.7 a	425.0 a	426.6 a	846.4 a	847.5 a	1333.1 a	1331.4 a	1492.9 a	1451.8 a	1454.0 a	1451.8 a
T_5_	160.0 ab	155.9 ab	383.4 a	387.6 abc	732.7 a	728.2 a	1209.0 a	1215.2 a	1309.5 a	1313.9 a	1325.0 a	1313.9 a
T_6_	186.2 a	172.2 a	428.3 a	425.6 ab	849.6 a	848.6 a	1335.9 a	1342.9 a	1493.3 a	1452.8 a	1453.7	1452.8 a
T_7_	160.7 ab	153.3 ab	384.9 a	380.6 abc	720.9 a	730.5 a	1208.3 a	1202.8 a	1300.6 a	1300.3 a	1320.9 a	1320.3 a
T_8_	176.0 a	173.1 a	417.9 a	441.1 a	840.8 a	845.2 a	1331.8 a	1325.2 a	1491.0 a	1450.8 a	1451.2 a	1450.8 a
T_9_	160.4 ab	154.0 ab	389.2 a	383.4 abc	721.6 a	753.4 a	1207.8 a	1202.5 a	1303.5 a	1300.1 a	1306.9 a	1310.1 a
T_10_	176.7 a	178.1 a	424.5 a	449.1 a	846.5 a	847.7 a	1329.3 a	1331.0 a	1495.0 a	1452.1 a	1457.8 a	1452.1 a
T_11_	161.0 ab	150.6 ab	389.8 a	384.9 abc	719.0 a	755.9 a	1209.3 a	1205.5 a	1302.7 a	1300.3 a	1325.0 a	1315.3 a
T_12_	132.7 b	130.1 b	321.0 a	317.0 bc	450.8 b	445.6 b	564.3 b	560.3 b	587.0 b	591.8 b	582.1 b	591.8 b
F-test	*	*	*	**	*	*	*	*	*	*	*	*
SEm (±)	5.2	4.8	9.6	13.0	37.3	39.8	51.6	55.7	62.2	56.3	62.5	59.3
CV (%)	6.83	7.75	14.10	9.21	7.79	8.05	7.20	5.47	8.35	7.12	8.14	8.31

Treatment details of the *Kharif* and *Boro* rice are mentioned in Table 3. CV (%) = coefficient of variation; ** and * significant at *p* ≤ 0.01 and *p* ≤ 0.05, respectively; NS = not significant; different letters within the continuous columns indicate significant differences at the 1% level of probability.

**Table 5:** Effect of nutrient management on the leaf area index of rice at different growth stages.

Treatment	Leaf Area Index (LAI)									
	20 DAT		40 DAT		60 DAT		80 DAT		100 DAT	
	2014	2015	2014	2015	2014	2015	2014	2015	2014	2015
***Kharif* rice**										
T_1_	2.66	2.66	3.07	3.09	4.87	4.88	2.71	2.70	1.44	1.33
T_2_	2.66	2.64	3.05	3.04	4.86	4.85	2.69	2.68	1.42	1.30
T_3_	1.72	1.39	2.11	1.84	3.85	3.82	1.67	1.71	0.43	0.29
T_4_	2.65	2.62	3.04	3.05	4.85	4.86	2.70	2.70	1.40	1.30
T_5_	2.61	2.61	3.00	2.99	4.83	4.84	2.70	2.66	1.33	1.22
T_6_	2.64	2.62	3.05	3.01	4.86	4.87	2.70	2.69	1.42	1.24
T_7_	2.58	2.59	2.98	3.01	4.82	4.84	2.64	2.66	1.32	1.18
T_8_	2.62	2.61	3.02	2.99	4.87	4.88	2.64	2.71	1.40	1.20
T_9_	1.76	1.76	2.17	2.19	3.97	3.98	1.81	1.80	0.54	0.43
T_10_	2.65	2.64	3.05	3.06	4.87	4.88	2.69	2.69	1.42	1.27
T_11_	2.56	2.54	2.97	2.97	4.85	4.87	2.67	2.70	1.36	1.24
T_12_	1.61	1.36	1.95	1.74	3.52	3.56	1.35	1.38	0.20	0.05
F-test	NS	NS	NS	NS	NS	NS	NS	NS	NS	NS
SEm (±)	0.04	0.07	0.06	0.08	0.08	0.1	0.07	0.08	0.03	0.03
CV (%)	45.49	46.78	39.11	39.82	23.79	23.79	0.2	45.02	86.57	84.88
***Boro* rice**										
T_1_	2.83	2.74	3.38	3.45	5.54	5.53	3.43	3.40	2.91	2.95
T_2_	2.68	2.70	3.40	3.42	5.51	5.50	3.40	3.41	2.89	2.91
T_3_	1.70	1.66	2.45	2.41	4.48	4.41	2.38	2.43	1.91	1.89
T_4_	2.70	2.68	3.40	3.44	5.51	5.45	3.42	3.44	2.80	2.91
T_5_	2.60	2.61	3.34	3.37	5.51	5.43	3.41	3.46	2.77	2.88
T_6_	2.74	2.72	3.44	3.45	5.52	5.48	3.41	3.47	2.78	2.88
T_7_	2.68	2.69	3.40	3.40	5.43	5.48	3.36	3.38	2.78	2.79
T_8_	2.74	2.76	3.44	3.45	5.44	5.48	3.36	3.57	2.89	2.88
T_9_	1.90	1.84	2.48	2.55	4.64	4.63	2.53	2.50	2.01	2.05
T_10_	2.70	2.71	3.42	3.42	5.50	5.49	3.40	3.45	2.83	2.89
T_11_	2.69	2.70	3.41	3.44	5.45	5.47	3.38	3.43	2.77	2.84
T_12_	1.59	1.56	2.36	2.33	4.14	4.04	1.96	1.94	1.54	1.52
F-test	NS	NS	NS	NS	NS	NS	NS	NS	NS	NS
SEm (±)	0.06	0.05	0.04	0.04	0.1	0.1	0.06	0.07	0.09	0.08
CV (%)	44.41	44.41	34.66	34.39	20.94	21.01	34.94	34.54	42.53	41.72

The treatment details for *Kharif* and *Boro* rice are mentioned in Table 3. CV (%) = coefficient of variation; NS = not significant.

**Table 6:** Effect of nutrient management on tillers (m^−2^) of rice at different growth stages.

Treat	Tillers (m^−2^)											
	20 DAT		40 DAT		60 DAT		80 DAT		100 DAT		120 DAT	
	2014	2015	2014	2015	2014	2015	2014	2015	2014	2015	2014	2015
***Kharif* rice**												
T_1_	298.8 a	299.7 a	311.2	315.8 a	340.3	340.7 a	327.8 a	326.8	324.7 a	310.4	317.3 a	306.8
T_2_	296.9 a	292.1 a	300.3	308.8 a	330.2	333.3 a	305.3 ab	312	303.6 ab	298.3	298.6 ab	295.2
T_3_	263.1 a	228.6 a	278.8	274.1 ab	280.2	295.7 a	270.5 ab	286.3	263.1 ab	259.4	257.8 ab	259.1
T_4_	289.1 a	290.8 a	290.3	298.1 a	331.7	333.3 a	302.9 ab	312.7	302.5 ab	294.3	300.2 ab	291.6
T_5_	263.6 a	265.2 a	280.1	284.9 ab	280.3	288.5 ab	281.2 ab	280.2	280.9 ab	275.7	280.3 ab	274.0
T_6_	290.1 a	292.2 a	296.3	301.1 a	322.6	330.0 a	319.2 a	307.8	313.1 a	301.2	310.2 a	296.4
T_7_	265.0 a	264.0 a	280.5	282.2 ab	282.3	283.7 ab	280.7 ab	299.8	278.9 ab	281.2	271.5 ab	277.4
T_8_	291.8 a	293.3 a	296.3	306.2 a	337.3	340.1 a	318.7 a	320.2	312.1 a	297.7	309.3 a	296.0
T_9_	263.3 a	267.0 a	273.2	283.5 ab	280.8	287.0 ab	285.0 ab	302.5	280.5 ab	284.0	274.7 ab	274.1
T_10_	287.3 a	293.7 a	290.1	294.2 ab	325.5	325.3 a	313.0 a	309.5	309.6 a	296.6	305.3 a	293.1
T_11_	280.2 a	285.1 a	281.5	290.4 ab	281.9	301.7 a	282.0 ab	299.4	280.3 ab	276.3	275.2 ab	271.3
T_12_	198.3 b	151.3 b	206.0	205.7 b	212.7	210.7 b	208.7 b	208.0	204.5 b	200.8	201.7 b	195.9
F-test	*	*	NS	*	NS	**	**	NS	**	NS	**	NS
SEm (±)	7.7	10.2	9.4	9.6	9.6	12.7	10.5	10.4	8.7	8.4	11.2	9.8
CV (%)	13.49	9.54	13.65	10.53	15.50	9.31	11.84	18.94	11.98	23.91	12.16	12.42
***Boro* rice**												
T_1_	340.8 a	346.8 a	361.2	362.7 a	386.3 a	388.3 a	380.8 a	365.7	366.7 a	366.0	361.3 a	360.3 a
T_2_	326.9 a	325.1 a	330.3	335.1 a	383.6 a	383.8 a	375.3 a	372.3	368.6 a	363.0	360.0 a	361.8 a
T_3_	293.1 a	308.6 a	310.8	316.3 a	342.0 ab	340.3 ab	330.5 ab	331.7	321.1 a	325.7	300.8 ab	320.3 ab
T_4_	332.1 a	325.8 a	339.3	336.7 a	371.7 a	373.6 a	362.5 a	367.3	359.9 a	366.0	357.2 a	353.0 a
T_5_	299.6 a	323.0 a	330.1	330.3 a	345.3 ab	337.4 ab	331.2 ab	328.5	326.9 a	323.5	320.3 ab	320.3 ab
T_6_	337.1 a	332.2 a	346.3	348.3 a	362.6 a	372.9 a	359.2 a	370.0	353.1 a	369.2	347.2 a	362.5 a
T_7_	303.0 a	320.0 a	331.0	330.7 a	350.3 ab	343.3 a	330.7 ab	330.7	325.9 a	326.5	321.5 ab	323.3 ab
T_8_	338.8 a	333.3 a	340.3	341.3 a	383.3 a	373.1 a	358.7 a	363.6	352.1 a	361.3	350.3 a	352.7 a
T_9_	303.3 a	307.0 a	313.2	322.2 a	342.0 ab	346.3 ab	338.6 ab	332.0	331.5 a	326.5	321.7 ab	323.5 ab
T_10_	327.3 a	333.7 a	328.1	330.3 a	365.5 a	373.5 a	353.0 a	365.3	350.6 a	363.5	345.3 a	356.9 a
T_11_	309.2 a	319.0 a	323.5	325.0 a	349.9 ab	345.9 ab	342.0 a	330.7	335.3 a	322.2	315.2 ab	320.3 ab
T_12_	208.3 b	211.3 b	226.0	221.7 b	252.7 b	257.3 b	245.7 b	240.7	233.5 b	228.0	229.7 b	223.3 b
F-test	*	*	NS	*	**	**	**	NS	*	NS	**	**
SEm (±)	10.2	11.3	9.9	10.4	12.2	13.9	12	11.4	10.2	10	13.2	10.8
CV (%)	7.46	8.12	16.50	9.30	9.44	10.90	9.37	17.68	8.56	22.52	9.83	12.30

The treatment details for the *Kharif* and *Boro* rice are mentioned in Table 3. CV (%) = coefficient of variation; ** and * significant at *p* ≤ 0.01 and *p* ≤ 0.05, respectively; NS = not significant; different letters within the continuous columns indicate significant differences at the 1% level of probability.

**Table 7:** Effect of nutrient management on the yield attributes of rice.

Treatments	Yield Attributes of Rice									
	Panicles m^−2^		Grains Panicle^−1^		Spikelets Panicle^−1^		Test Weight (g)		Panicle Length (cm)	
	2014	2015	2014	2015	2014	2015	2014	2015	2014	2015
***Kharif* rice**										
T_1_	246.1 a	249.8	131.0 a	133.1	148.3 a	151.0	23.5 a	24.2	30.0	31.2
T_2_	241.1 b	244.8	127.0 b	128.2	146.5 ab	146.3	22.6 a	22.8	28.7	29.7
T_3_	200.2 f	219.7	110.0 d	110.5	133.0 ef	132.1	22.5 a	22.3	26.0	22.0
T_4_	245.0 a	245.9	120.0 c	124.5	143.0 c	148.0	22.8 a	23.2	29.9	30.9
T_5_	221.1 b	220.2	111.5 d	112.5	135.1 de	135.5	22.2 a	22.6	26.4	26.8
T_6_	232.7 c	236.5	125.2 b	128.6	147.5 ab	150.9	21.8 a	23.8	28.2	29.9
T_7_	220.2 d	221.0	112.1 d	114.8	136.5 d	135.1	22.6 a	23.6	26.3	26.1
T_8_	245.0 a	247.0	122.2 c	127.6	147.3 ab	148.8	22.5 a	23.2	29.3	30.6
T_9_	220.2 d	214.2	112.1 d	113.2	136.2 d	135.0	22.2 a	23.5	26.2	26.7
T_10_	245.0 a	245.1	111.0 d	125.4	144.9 bc	146.1	22.2 a	23.2	27.0	30.4
T_11_	220.6 d	220.2	112.1 d	114.0	131.3 fg	136.0	22.2 a	23.0	25.0	25.7
T_12_	212.3 e	204.0	65.5 e	61.8	129.7 g	127.1	20.0 b	19.7	21.0	19.1
F-test	**	NS	**	NS	**	NS	*	NS	NS	NS
SEm (±)	8.8	9.8	6.4	6.1	3.9	5.1	1.7	1	1.1	1.4
CV (%)	24.9	28.7	18.8	17.8	11.4	14.9	4.9	2.9	3.4	4.1
***Boro* rice**										
T_1_	281.0	282.2	140.0	145.4	155.1	159.4	24.0	25.6	32.5	33.2
T_2_	269.7	280.4	133.0	136.0	151.0	152.2	22.9	22.8	29.9	30.3
T_3_	239.8	234.7	90.0	91.5	126.2	125.1	21.0	20.3	26.6	25.3
T_4_	260.4	274.3	125.0	140.1	154.9	156.1	23.3	23.5	31.4	33.1
T_5_	233.9	236.4	121.2	120.0	131.3	136.0	22.7	22.6	26.4	27.1
T_6_	277.6	279.5	130.0	132.3	155.0	158.0	22.3	23.8	30.1	32.3
T_7_	232.5	237.7	120.0	121.1	130.4	131.7	23.1	23.4	29.0	28.7
T_8_	278.0	281.4	134.9	136.3	150.6	153.0	23.0	23.2	31.5	32.1
T_9_	232.0	235.2	120.0	122.0	134.4	137.9	23.8	24.9	22.4	24.1
T_10_	267.0	268.1	137.9	138.2	151.1	155.3	22.6	23.6	25.5	27.2
T_11_	236.6	230.2	121.6	121.2	130.7	131.0	22.0	23.1	24.9	26.6
T_12_	215.3	210.0	80.0	76.0	121.0	120.0	20.2	20.0	22.8	20.3
F-test	NS	NS	NS	NS	NS	NS	NS	NS	NS	NS
SEm (±)	8	8.5	6.2	6.9	6	5.9	1.8	1	1	1.4
CV (%)	23.4	25.1	18.1	20.1	17.6	17.3	5.2	2.8	2.8	4.0

The treatment details for *Kharif* and *Boro* rice are mentioned in Table 3. CV (%) = coefficient of variation; ** and * significant at *p* ≤ 0.01 and *p* ≤ 0.05, respectively; NS = not significant; different letters within the continuous columns indicate significant differences at the 1% level of probability.

**Table 8:** Effect of nutrient management on the nutrient uptake (kg ha^−1^) of *Kharif* rice.

Treatment	N Uptake in Grain		N Uptake in Straw		P Uptake in Grain		P Uptake in Straw		K Uptake in Grain	
	2014	2015	2014	2015	2014	2015	2014	2015	2014	2015
T_1_	68.5	71.7	29.6	31.9	19.3	21.0	11.8	14.1	23.6	24.9
T_2_	55.7	61.8	25.2	27.4	17.3	18.5	10.4	13.3	20.9	22.8
T_3_	30.9	28.7	9.7	11	16.1	15.3	10.2	9.7	19.3	14.2
T_4_	64.3	65.5	27.7	28.5	16.5	18.4	10.2	12.2	21.6	22.1
T_5_	64.3	64.8	28.4	29.7	16.0	15.5	8.8	9.2	20.5	20.9
T_6_	65.9	65.6	28.4	29.1	17.7	19.2	11.2	12.5	16.4	16.9
T_7_	65.3	63.8	27.9	29.8	16.9	18.4	10.9	13.2	10.1	9.1
T_8_	64.5	65.7	27.9	30.8	17.4	18.5	11.3	13.2	22.4	22.7
T_9_	66.4	63.7	27.7	29.8	16.8	18.0	11.8	13.3	20.9	22.8
T_10_	66.4	67	27.5	30.3	17.0	18.7	11.5	13.1	21.7	22.8
T_11_	65.5	66.5	27.5	30.3	17.9	18.4	11.8	13.3	22.4	22.4
T_12_	20.8	16.4	14.3	9.4	7.57	6.45	4.5	4.6	4.34	3.11
F-test	NS	NS	NS	NS	NS	NS	NS	NS	NS	NS
SEm (±)	1.33	2.26	0.83	0.83	0.8	0.83	0.49	0.48	1.2	0.9
CV (%)	3.91	6.63	2.44	2.43	2.50	2.40	1.40	1.41	3.5	2.65
Treatment	K uptake in straw		Zn uptake in grain		Zn uptake in straw		S uptake in grain		S uptake in straw	
	2014	2015	2014	2015	2014	2015	2014	2015	2014	2015
T_1_	47.8	47.9	0.13	0.14	0.29	0.30	6.7	7.0	3.1	3.2
T_2_	43.0	45.0	0.12	0.12	0.26	0.25	6.6	6.5	2.9	3.0
T_3_	32.1	32.0	0.07	0.06	0.18	0.17	3.4	3.7	1.7	1.8
T_4_	45.9	47.1	0.13	0.11	0.27	0.26	6.4	6.5	2.7	3.0
T_5_	45.4	44.8	0.10	0.10	0.25	0.23	6.0	6.1	2.5	2.8
T_6_	32.8	36.2	0.12	0.11	0.27	0.28	6.3	6.6	2.8	3.1
T_7_	30.0	38.4	0.11	0.13	0.26	0.29	6.5	6.6	2.9	3.0
T_8_	45.5	45.7	0.10	0.10	0.20	0.18	6.6	6.9	2.9	2.9
T_9_	44.5	45.4	0.09	0.08	0.13	0.10	6.6	6.5	2.8	3.0
T_10_	45.1	45.8	0.11	0.12	0.26	0.26	5.7	5.6	2.3	2.5
T_11_	40.8	45.7	0.11	0.11	0.28	0.27	5.3	4.4	2.3	2.1
T_12_	10.9	10.5	0.04	0.03	0.12	0.11	1.98	1.72	1.12	1.1
F-test	NS	NS	NS	NS	NS	NS	NS	NS	NS	NS
SEm (±)	1.67	1.92	0.004	0.009	0.01	0.009	0.14	0.19	0.1	0.1
CV (%)	4.89	5.64	0.012	0.027	0.028	0.027	0.41	0.56	0.28	0.29

The treatment details for *Kharif* and *Boro* rice are mentioned in Table 3. CV (%) = coefficient of variation; NS = not significant.

**Table 9:** Effect of nutrient management on the nutrient uptake (kg ha^−1^) of *Boro* rice.

Treatments	N Uptake in Grain		N Uptake in Straw		P Uptake in Grain		P Uptake in Straw		K Uptake in Grain	
	2014–2015	2015–2016	2014–2015	2015–2016	2014–2015	2015–2016	2014–2015	2015–2016	2014–2015	2015–2016
T_1_	98.9	107.7	57.5	56.4	34.3	38.3 a	28.9 a	30.2 a	28.5	33.0 a
T_2_	76.7	81.1	41.2	51.5	28.5	30.8 f	25.3 de	25.8 ef	27.0	30.5 f
T_3_	17.3	15.9	18.3	14.2	17.9	17.3 h	15.0 h	17.2 h	15.0	16.3 h
T_4_	87.6	100.5	52.4	54.1	25.5	27.8 g	20.8 g	24.3 g	27.4	30.8 d
T_5_	86.6	99.9	57.2	56.2	15.3	12.8 i	10.2 i	7.0 i	27.7	30.7 de
T_6_	90.1	99.3	55.4	54.4	31.0	33.6 e	24.8 ef	25.3 f	19.9	25.1 g
T_7_	87.7	97.2	53.2	52.2	30.6	33.7 d	25.8 cd	26.9 cd	12.1	10.0 i
T_8_	88.1	102.4	52.3	54.5	32.1	33.7 d	26.1 c	28.0 b	27.0	31.0 c
T_9_	88.7	98.6	52.1	53.6	32.0	34.0 b	24.7 ef	26.4 de	26.9	30.6 ef
T_10_	87.3	100.3	52.4	55.3	32.0	33.8 c	27.5 b	27.7 bc	26.6	30.5 f
T_11_	88.7	95.5	51.4	51.1	31.1	33.8 c	24.2 f	26.6 de	27.9	31.9 b
T_12_	6.3	12.4	16.9	11	4.86	4.16 j	4.29 j	3.45 j	2.92	2.62 j
F-test	NS	NS	NS	NS	NS	**	**	**	**	**
SEm (±)	3.5	3.6	1.7	1.9	1.92	1.67	1.67	2.46	0.67	0.83
CV (%)	11.7	10.2	4.9	5.7	5.64	4.89	4.89	7.22	1.96	2.44
Treatments	K uptake in straw		Zn uptake in grain		Zn uptake in straw		S uptake in grain		S uptake in straw	
	2014–2015	2015–2016	2014–2015	2015–2016	2014–2015	2015–2016	2014–2015	2015–2016	2014–2015	2015–2016
T1	53.3 a	64.3 a	0.19	0.19	0.35 a	0.34 ab	9.2 a	9.3 a	6.4 a	6.7 a
T_1_	48.5 d	59.5 c	0.18	0.18	0.32 a	0.33 ab	8.6 ab	8.6 ab	6.4 a	6.3 abc
T_2_	37.7 f	36.2 h	0.12	0.14	0.20 bc	0.21 bc	4.7 d	4.7 d	4.2 d	4.5 d
T_3_	51.6 b	63.4 b	0.18	0.18	0.31 a	0.34 ab	8.6 ab	8.6 ab	6.2 ab	6.5 ab
T_4_	48.7 cd	56.9 e	0.16	0.16	0.27 ab	0.32 ab	8.2 b	8.5 ab	5.6 bc	5.7 c
T_5_	37.0 f	41.2 g	0.18	0.18	0.31 a	0.32 ab	8.6 ab	8.7 ab	6.0 ab	6.2 abc
T_6_	34.9 g	51.0 f	0.18	0.18	0.33 a	0.35 a	8.9 ab	8.8 ab	6.0 ab	6.3 abc
T_7_	48.6 d	63.8 ab	0.13	0.12	0.22 b	0.23 abc	8.8 ab	8.8 ab	6.5 a	6.3 abc
T_8_	45.5 e	58.6 d	0.10	0.09	0.11 d	0.10 c	8.3 b	8.4 b	5.0 c	5.9 bc
T_9_	50.7 b	56.8 e	0.18	0.18	0.34 a	0.34 ab	6.8 c	6.9 c	3.1 e	3.5 e
T_10_	49.6 c	56.8 e	0.18	0.18	0.34 a	0.34 ab	4.9 d	4.6 d	2.0 f	2.1 f
T_11_	12.6 h	11.98 i	0.03	0.02	0.12 cd	0.11 c	1.62 e	1.51 e	1.23 g	1.08 g
F-test	**	**	NS	NS	**	**	**	**	**	**
SEm (±)	1.8	2.67	0.006	0.007	0.012	0.01	0.23	0.25	0.15	0.17
CV (%)	5.28	7.82	0.018	0.021	0.037	0.03	0.69	0.75	0.43	0.49

The treatment details for *Kharif* and *Boro* rice are mentioned in Table 3. CV (%) = coefficient of variation; ** and * significant at *p* ≤ 0.01 and *p* ≤ 0.05, respectively; NS = not significant; different letters within the continuous columns indicate significant differences at the 1% level of probability.

**Table 10:** Effect of nutrient management on the nutrient balance of soil after the second year of *Boro* rice.

Treatment	Initial Soil Status (kg/mg ha^−1^)					The 2nd Year of *Boro* Rice (kg/mg ha^−1^)					Nutrient Balance (kg/mg ha^−1^)				
	N	P	K	Zn	S	N	P	K	Zn	S	N	P	K	Zn	S
T_1_	230.5	11.2	125.2	0.2	10.5	290.5	31.7	211.2	0.6	27.2	60.0	20.5	86.0	0.4	16.7
T_2_	230.5	11.2	125.2	0.2	10.5	242.3	27.9	209.2	0.5	24.9	11.8	16.7	84.0	0.3	14.4
T_3_	230.5	11.2	125.2	0.2	10.5	216.4	28.5	208.3	0.4	24.5	−14.1	17.3	83.1	0.2	14.0
T_4_	230.5	11.2	125.2	0.2	10.5	286.3	26.6	210.2	0.5	25.5	55.8	15.4	85.0	0.3	15.0
T_5_	230.5	11.2	125.2	0.2	10.5	286.6	9.0	207.8	0.5	24.4	56.1	−2.2	82.6	0.2	13.9
T_6_	230.5	11.2	125.2	0.2	10.5	285.8	30.3	196.0	0.6	24.4	55.3	19.1	70.8	0.3	13.9
T_7_	230.5	11.2	125.2	0.2	10.5	282.8	28.6	112.1	0.6	24.1	52.3	17.4	−13.1	0.3	13.6
T_8_	230.5	11.2	125.2	0.2	10.5	282.7	28.3	207.8	0.4	24.2	52.2	17.1	82.6	0.2	13.7
T_9_	230.5	11.2	125.2	0.2	10.5	280.7	28.7	204.9	0.1	25.0	50.2	17.5	79.7	−0.1	14.5
T_10_	230.5	11.2	125.2	0.2	10.5	284.2	29.0	202.4	0.5	16.2	53.7	17.8	77.2	0.3	5.7
T_11_	230.5	11.2	125.2	0.2	10.5	276.0	27.2	201.9	0.4	3.4	45.5	16.0	76.7	0.2	−7.1
T_12_	230.5	11.2	125.2	0.2	10.5	214.3	8.8	112.4	0.1	3.2	−16.2	−2.4	−12.8	−0.1	−7.3
STDEV	0.00	0.00	0.00	0.00	0.00	27.99	7.81	36.73	0.17	8.49	27.99	7.81	36.7	0.16	8.49
±SEm	0.00	0.00	0.00	0.00	0.00	8.08	2.26	10.60	0.05	2.45	8.08	2.26	10.6	0.05	2.45

The treatment details for the *Kharif* and *Boro* rice are mentioned in Table 3.

## Data Availability

Most of the data are available in all the tables and figures of the manuscript.

## References

[1] Deep M., Kumar R.M., Saha S., Singh A. (2018). Rice-based cropping systems. Indian Farming.

[2] Lal B., Gautam P., Panda B.B., Raja R., Singh T., Tripathi R., Shahid M., Nayak A.K. (2017). Crop and varietal diversification of rainfed rice based cropping systems for higher productivity and profitability in Eastern India. PLoS ONE.

[3] Bhatt R., Kukal S.S., Busari M.A., Arora S., Yadav M. (2016). Sustainability issues on rice—Wheat cropping system. Int. Soil Water Conserv. Res..

[4] Jat M.L., Majumdar K., McDonald A., Sikka A.K., Paroda R.S. Book of extended summaries. National Dialogue on Efficient Nutrient Management for Improving Soil Health. Proceedings of the TAAS, ICAR, CIMMYT, IPNI, CSISA, FAI.

[5] Jata R.A., Dungranib R.A., Arvadiab M.K., Sahrawatc K.L. (2012). Diversification of rice (*Oryza sativa* L.) based cropping systems for higher productivity, resource-use efficiency and economic returns in south Gujarat, India. Arch. Agron. Soil Sci..

[6] Ye T., Li Y., Zhang J., Hou W., Zhou W., Lu J., Xing Y., Li X. (2019). Nitrogen, phosphorus, and potassium fertilization affects the flowering time of rice (*Oryza sativa* L.). Glob. Ecol. Conserv..

[7] Di Mola I., Ottaiano L., Cozzolino E., Senatore M., Giordano M., El-Nakhel C., Sacco A., Rouphael Y., Colla G., Mori M. (2019). Plant-based biostimulants influence the agronomical, physiological, and qualitative responses of baby rocket leaves under diverse nitrogen conditions. Plants.

[8] Fageria N.K., Baligar V.C. (2013). Methodology for evaluation of lowland rice genotypes for nitrogen use efficiency. J. Plant Nutr..

[9] Mori A., Fukuda T., Vejchasarn P., Nestler J., Pariasca-Tanaka J., Wissuwa M. (2016). The role of root size versus root efficiency in phosphorus acquisition in rice. J. Exp. Bot..

[10] Cakmak I. (2005). The role of potassium in alleviating detrimental effects of abiotic stresses in plants. J. Plant Nutr. Soil Sci..

[11] Patel P.K., Kadivala V.A.H., Patel V.N. (2019). Role of Sulphur in Oilseed Crops: A Review. J. Plant Dev. Sci..

[12] Impa S.M., Johnson-Beebout S.E. (2012). Mitigating zinc deficiency and achieving high grain Zn in rice through integration of soil chemistry and plant physiology research. Plant Soil.

[13] Singh M.V., Alloway B.J. (2008). Micronutrient Deficiencies in Crops and Soils in India. Micronutrient Deficiencies in Global Crop Production.

[14] Sharma A., Patni B., Shankhdhar D., Shankhdhar S.C. (2013). Zinc—An indispensable micronutrient. Physiol. Mol. Biol. Plants.

[15] Prasad R., Shivay Y.S., Kumar D. (2014). Agronomic biofortification of cereal grains with iron and zinc. Adv. Agron..

[16] Singh V.K., Dwivedi B.S., Mishra R.P., Shukla A.K., Timsina J., Upadhyay P.K., Shekhawat K., Majumdar K., Panwar A.S. (2019). Yields, Soil Health and Farm Profits under a Rice-Wheat System: Long-Term Effect of Fertilizers and Organic Manures Applied Alone and in Combination. Agronomy.

[17] Majumdar K., Jat M.L., Pampolino M., Satyanarayana T., Dutta S., Kumar A. (2013). Nutrient management in wheat: Current scenario, improved strategies and future research needs in India. J. Wheat Res..

[18] Shahane A.A., Shivay Y.S., Prasanna R. (2020). Nutrient removal by rice–wheat cropping system as influenced by crop establishment techniques and fertilization options in conjunction with microbial inoculation. Sci. Rep..

[19] Sravan U.S., Ramana Murthy K.V. (2018). Enhancing Productivity in Rice-Based Cropping Systems, Plant Competition in Cropping Systems, Daniel Dunea.

[20] Ghosh S., Guchhait S.K., Hu X.F. (2015). Characterization and evolution of primary and secondary laterites in northwestern Bengal Basin, West Bengal, India. J. Palaeogeogr..

[21] Bouyoucos G.J. (1962). Hydrometer method improved for making particle size analysis of soils. J. Agron..

[22] Jackson M.L. (1973). Soil Chemical Analysis.

[23] Subbiah B.V., Asija G.L. (1956). A rapid procedure for the determination of available nitrogen in soils. Curr. Sci..

[24] Bray R.H., Kurtz L.T. (1945). Determinations of total, organic and available forms of phosphorus in soils. Soil Sci..

[25] Hanway J.J., Heidel H. (1952). Soil analyses methods as used in Iowa State College Soil Testing Laboratory. Iowa Agric..

[26] Lindsay W.L., Norvell W.A. (1978). Development of DTPA soil test for Zn, Fe, Mn and Cu. Soil Sci. Soc. Am. J..

[27] Chesnin L., Yien C.H. (1950). Turbid metric Determination of Available Sulphates. Soil Sci. Soc. Am. J..

[28] Watson D.J. (1952). The physiological basis of variation in yield. Adv. Agron..

[29] Tandon H.L.S. (2007). Soil Nutrient Balance Sheets in India: Importance, Status, Issues, and Concerns. Better Crop. India.

[30] Cochran W.G., Cox G.M. (1977). Experimental Design.

[31] Shankar T., Banerjee M., Malik G.C., Dutta S., Maiti D., Maitra S., Alharby H., Bamagoos A., Hossain A., Ismail I.A. (2021). The Productivity and Nutrient Use Efficiency of Rice-Rice-Black GramCropping Sequence Are Influencedby Location Specific Nutrient Management. Sustainability.

[32] Show R. (2007). Growth and Productivity of Boro Rice under Different Nitrogen and Water Regimes in Lateritic Belt of West Bengal. Ph.D. Thesis.

[33] Shekara B.G., Shreedhara D. (2010). Growth and yield of aerobic rice (*Oryza sativa* L.) influenced by different levels of NPK in Cauvery command area. J. Maharashtra Agric. Univ..

[34] Pandey N., Verma A.K., Tripathi R.S. (2001). Effect of planting time and nitrogen on tillering pattern, drymatter accumulation and grain yield of hybrid rice. Ind. J. Agric. Sci..

[35] Pariyani A.K., Naik K.R. (2004). Effect of nitrogen level and seedling number on yield attributes and yield of rice hybrid. J. Soils Crop..

[36] Hu R., Cao J., Huang J., Peng S., Zhong X., Zou Y., Yang J., Buresh R.J. (2007). Farmer participatory testing of standard and modified site-specific nitrogen management for irrigated rice in China. Agric. Sci..

[37] Huang J., He F., Cui K., Buresh R.J., Xu B., Gong W., Peng S. (2008). Determination of optimal nitrogen rate for rice varieties using a chlorophyll meter. Field Crop. Res..

[38] Mohapatra A.K. (2003). Studies on Direct and Residual Effect of Secondary and Micronutrients in Rice (Hybrid)—Rice Cropping Sequence. Ph.D. Thesis.

[39] Trivedi V.K., Pandey M.R., Pathak R.K., Kala D.C. (2015). Balanced use of nutrients for high crop yield and quality of rice in central uttarpradesh. Int. J. Tech. Res. Appl..

[40] Murthy K.M., Rao A.U., Vijay D., Sridhar T.V. (2015). Effect of levels of nitrogen, phosphorus and potassium on performance of rice. Indian J. Agric. Res..

[41] Yadav M.P., Tiwari U.S., Raj J. (2007). Studies on site specific nutrient management (SSNM) for maximization of yield and economics in hybrid rice (*Oryza sativa*). Plant Arch..

[42] Nath D.K., Haque F., Amin F., Islam M.S., Saleque M.A. (2013). Farmers’ participatory site specific nutrient management in gangetic tidal floodplain soil for high yielding *Boro* rice (*Oryza sativa* L.). Agriculturists.

[43] Singh B.B., Singh J., Singh G., Kaur G. (2015). Effects of Long Term Application of Inorganic and Organic Fertilizers on Soil Organic Carbon and Physical Properties in Maize—Wheat Rotation. Agronomy.

[44] Ranamukhaarachchi S.L., Ratnayake W.M. (2006). The effect of straw, stubble, and potassium on grain yield of rice in rice-rice cropping systems in the mid-country wet zone of Srilanka. Sci. Asia.

[45] Shankar T., Malik G.C., Banerjee M., Ghosh A. (2014). Nutrient optimization on growth and productivity of rice in the red and lateriticbelt of West Bengal. J. Crop Weed.

[46] Pampolinoa M.F., Manguiata I.J., Ramanathanb S., Ginesc H.C., Tand P.S., Chid T.T.N., Rajendrane R., Buresh R.J. (2007). Environmental impact and economic benefits of site specific nutrient management (SSNM) in irrigated rice systems. Agric. Syst..

[47] Chandrapala A.G., Yakadri M., Kumar R.M., Raj G.B. (2010). Productivity and economics of rice (*Oryza sativa*)—Maize (*Zea mays*) as influenced by methods of crop establishment, Zn and S application in rice. Indian J. Agron..

[48] Porpavai S., Devasenapathy P., Siddeswaran K., Jayaraj T. (2011). Impact of various rice based cropping systems on soil fertility. J. Cereals Oilseeds.

[49] Singh A.K., Meena M.K., Upadhyaya A. (2012). Effect of sulphur and zinc on rice performance and nutrient dynamics in plants and soil of Indo Gangetic plains. J. Agric. Sci..

[50] Deka A.M., Kalita H., Borah N., Zaman A.S.N. (2019). Nutrient uptake and nutrient balance as influenced by different rice based cropping patterns in Assam. J. Crop Weed.

[51] Panwar A.S., Shamim M., Babu S., Ravishankar N., Prusty A.K., Alam N.M., Singh D.K., Bindhu J.S., Kaur J., Dashora L.N. (2019). Enhancement in Productivity, Nutrients Use Efficiency, and Economics of Rice-Wheat Cropping Systems in India through Farmer’s Participatory Approach. Sustainability.

